# Influenza A and other respiratory viruses show distinct associations with acute respiratory presentations and temporal correlation with hospital admissions

**DOI:** 10.1099/jmm.0.002203

**Published:** 2026-09-16

**Authors:** May Husein, Muthanna Samara, M. Rami Alfarra, Ali Al-Kinani, Nader Al-Dewik, Peter V. Coyle

**Affiliations:** 1 Department of Laboratory Medicine and Pathology (DLMP), Molecular Virology Lab, Hamad General Hospital (HGH), Hamad Medical Corporation (HMC), Doha, Qatar; 2 Drug Discovery, Delivery and Patient Care Theme (DDDPC), Faculty of Science, Engineering and Computing, Kingston University London, Penrhyn Road, Kingston upon Thames, UK; 3 Department of Psychology, Kingston University London, Kingston upon Thames, UK; 4 Qatar Environment and Energy Research Institute, Hamad Bin Khalifa University, Doha, Qatar; 5 Department of Research, Women’s Wellness and Research Center, Hamad Medical Corporation, Doha, Qatar; 6 Interim Translational Research Institute (iTRI), Hamad Medical Corporation (HMC), Doha, Qatar; 7 Genomics and Precision Medicine (GPM), College of Health and Life Sciences (CHLS), Hamad Bin Khalifa University (HBKU), Doha, Qatar; 8 Biomedical Research Center, QU Health, Qatar University, Doha, Qatar; 9 Wellcome-Wolfson Institute for Experimental Medicine, Queens University, Belfast, UK

**Keywords:** acute respiratory infection, asthma, bronchiolitis, Influenza A, pneumonia

## Abstract

**Introduction.** Influenza A is the major cause of hospitalization due to acute respiratory virus infections (ARIs) worldwide, while other respiratory viruses are thought to play lesser roles. However, viral co-infection, host demographics and environmental factors, including air quality, may influence clinical presentation and hospital admissions. This study characterizes their clinical associations during the last influenza A outbreak in Qatar before the emergence of severe acute respiratory syndrome coronavirus 2.

**Gap statement.** The relative contributions of host demographics, viral co-infection and environmental factors to ARI hospital admissions remain incompletely understood.

**Aim.** To assess the association of host demographics, viral co-infection and air quality on influenza A admissions for pneumonia, bronchiolitis and reactive airway exacerbations.

**Methodology.** Between 1 November 2019 and 28 March 2020, 1,801 patients admitted with ARIs were classified using ICD-10 codes into bronchiolitis, reactive airway exacerbations and pneumonia. Respiratory samples were analysed by real-time PCR (RT-PCR) for influenza A and other common respiratory viruses, with non-influenza A grouped collectively as other respiratory viruses (ORVs). Air quality metrics at admission were ozone, sulphur dioxide, nitrogen dioxide, carbon monoxide, nitrogen oxide and fine particulate matter. Associations between influenza A, air quality, viral co-infection, age, gender and nationality were assessed.

**Results.** Distinct waves of influenza A and respiratory syncytial virus (RSV) were observed, while human rhinovirus (HRV) transmission persisted at a high level, with remaining viruses at low levels. Influenza A was significantly associated with pneumonia, while ORVs were the main causes of bronchiolitis (RSV) and reactive airway exacerbations (HRV); viral co-infections were uncommon. Bronchiolitis predominantly affected infants <12 months, whereas reactive airway exacerbations were seen across wider age bands. No consistent independent associations between air quality metrics and ARIs were observed after multivariable adjustment during the 5-month study period. Combining all the respiratory viruses showed a strong correlation with patterns of hospital admissions (*r*=0.88, 95% confidence interval: 0.72, 0.95).

**Conclusion.** Age and viral type were the most significant determinants of admission. While the associations of influenza A, HRV and RSV with pneumonia, acute exacerbations and bronchiolitis, respectively, were confirmed, all viruses had similar associations, and the strongest correlation was observed when all viruses were treated as a single wave.

## Data Summary

This is a research article and all data generated or analysed during this study are included in this published article. All enquiries should be directed to the corresponding authors.

## Introduction

The respiratory tract is a complex ecological environment, where co-infections and microbial interactions can result in respiratory diseases [1], with air quality and meteorological conditions thought to be important cofactors [2, 3]. In a recent study in Qatar, young children, rather than meteorological factors, served as the primary determinant of viral transmission [4]. Lower respiratory tract infections (LRIs) are the fourth leading cause of death globally, affecting people of all ages and causing some 2 million deaths annually, in both temperate and hot climates; in Saudi Arabia, they were reported in over 15% of the population each year [5] with a similar incidence reported in Qatar [6]. A recent study from Qatar confirmed that bacterial LRIs in hospitalized patients were significantly more likely to have a respiratory virus co-infection [7], indicating that viruses are common triggers of pneumonia, even in a hospital setting. Acute respiratory infections (ARIs) are therefore multifactorial, influenced by viral types, host characteristics, lifestyle choices and the environment [8]. Infants and children under 2 years have a heightened risk of hospitalization, with RSV the principal cause of bronchiolitis in children under 6 months, which may also increase the risk of later asthma development [9]. Older adults, especially in economically disadvantaged areas, have higher rates of emergency room visits and hospital admissions for pneumonia, acute exacerbation of asthma (AE-asthma) and acute exacerbation of chronic obstructive pulmonary disease (AE-COPD). Hawker *et al*. [10] reported that socioeconomic disadvantage raises the rates of hospitalization for respiratory infections, with extremes of age over-represented [11]. Age, smoking during pregnancy, poor educational background and a history of asthma have been associated with a higher risk of ARI in children [12] and severe influenza and pneumococcal disease [13, 14].

This study aimed to describe the winter impact of influenza A (Flu A) on hospital admissions in Qatar and to evaluate the associations with respiratory viral co-infections, host demographic factors (age, gender and nationality) and air quality metrics. Clinical categories of pneumonia, bronchiolitis and reactive airway exacerbations based on ICD-10 codes were used during the study period that ran from 1 November 2019 to 28 March 2020, prior to the emergence of severe acute respiratory syndrome coronavirus 2 (SARS-CoV-2).

## Methods

### Study design and data collection

Respiratory virus and atypical results from 1 November 2019 to 28 March 2020 from 1,801 patient admission episodes with ARI were classified into three groups using ICD-10 codes. The groups were reactive airway disease, pneumonia and bronchiolitis. Respiratory specimens, including upper and/or lower respiratory tract samples as clinically indicated, were tested using the Fast Track Diagnostics Respiratory Pathogens 21 multiplex real-time (PCR) RT-PCR assay (FTD™, Luxembourg). Results were interpreted according to the manufacturer’s criteria and laboratory quality-control procedures. Detection of a single pathogen was classified as mono-infection, whereas simultaneous detection of two or more respiratory viruses was classified as co-infection. The samples came from hospital in-patients, adult and paediatric emergency departments, primary healthcare centres and outpatient clinics. The respiratory tests included Flu A virus, influenza B virus, human rhinovirus (HRV), human coronaviruses 229E, NL63, HKU1 and OC43, human parainfluenza viruses 1 through 4, human metapneumoviruses, human bocavirus, *Mycoplasma pneumoniae*, respiratory syncytial virus (RSV) and human adenovirus. Patients were included if they were hospitalized and had a respiratory viral screen within 7 days of admission. Only the first test per patient was analysed to avoid duplicates. Cases were excluded if environmental data for that period were unavailable. A bigger cohort that included screening results inclusive of >7 days was used to calculate an overall correlation between hospital admission and any respiratory virus infection.

### Demographic data and environmental factors

Average weekly ambient air quality metrics included carbon monoxide (CO), nitrogen oxide (NO), nitrogen dioxide (NO_2_), sulphur dioxide (SO_2_), O_3_, and PM_2.5_. These were recorded at around 14 meters above sea level at a latitude of 25°16′45″ N and longitude of 51°31′20″ E. The daily incidence of each pathogen, as detected by RT- PCR, and associations with air quality metrics, were analyzed using the average weekly means of the respective ambient air quality metrics. The study population comprised 1,801 patient episodes, consisting of 1,113 males and 688 females, with a male-to-female ratio of 1.62:1. The participants were categorized into eight age groups: <1 year (infants), 1–4 years (preschool age), 5–12 years (school age), 13–18 years (adolescence), 19–30 years (young adults), 31–45 years (adults), 46–60 years (older adults) and >60 years (elderly). Qatari nationals made up 34.6%, with the remaining 65.4% primarily from other Arab and Asian countries (Egypt, Syria, Jordan, Gulf States, India, Bangladesh, the Philippines and Nepal) with smaller contributions from other nationalities. To facilitate statistical analysis and interpretation, continuous air quality data were transformed into categorical variables by dichotomizing each factor into two levels (low and high). The threshold values were determined based on the mean concentrations observed in the dataset. Specifically, O_3_ levels were categorized as low (≤20.84 ppb) and high (>20.84 ppb), PM_2.5_ as low (≤27.68 84 µg m^−3^) and high (>27.69 84 µg m^−3^), SO_2_ as low (≤3.48 ppb) and high (>3.48 ppb), NO_2_ as low (≤31.32 ppb) and high (>31.32 ppb) and NO as low (≤21.41 ppb) and high (>21.41 ppb), and CO was classified as low (≤0.62 ppm) and high (>0.62 ppm). It is worth noting that these value thresholds were based on weekly means of data collected during the study period.

Age reference groups for each ARI were defined based on categories showing a low observed severity or hospital admission risk. This approach improves interpretability by comparing odds ratios (ORs) against a lowest-risk baseline. For the acute exacerbation group, infants aged <1 year were selected since AE-asthma and AE-COPD are rare in this age group but increase at age 1–4 and >65 years, respectively [15]. For pneumonia, adults aged 19–30 years were chosen, reflecting the lowest hospitalization rates [16]. For bronchiolitis, children aged 1–4 years served as the reference, highlighting the increased risk among infants <1 year [17].

### Statistical analysis

Statistical analysis was conducted using IBM SPSS 29 software (SPSS, Chicago, IL, USA). Categorical and binary variables were expressed as counts and percentages. The overall incidence of respiratory viral and atypical infections, including Flu A, along with gender, age, nationality and air quality metrics, was evaluated using chi-square analysis. This analysis aimed to investigate the differences in positive and negative confirmed infections across the three ARI groups – bronchiolitis, reactive airway exacerbations and pneumonia. All other viruses, excluding Flu A, were in the other respiratory virus (ORV) group.

Regression analyses were performed to assess the association of gender, age, nationality and air quality metrics with patients admitted for ARI. Results were reported as ORs with 95% confidence intervals (CIs) and statistical significance was defined as two-sided *P*<0.05. Multicollinearity was assessed using the Variance Inflation Factor (VIF), and variables with VIF values <5 were retained, indicating no significant multicollinearity and only moderate correlations among the predictors. Univariate logistic regression was used to assess the association between air quality metrics (CO, NO, NO_2_, SO_2_, O_3_ and PM_2.5_) and ARI. Separate univariate logistic regression analyses were performed for patients admitted with respiratory infection, including Flu A. After performing univariate regression, all air quality metrics were retained in the multivariable models due to their potential to confound associations with viral co-infections, given their seasonal overlap and intercorrelated patterns. Two multivariable logistic regression models were developed to investigate the association between air quality, viral co-infections and ARI admissions. Model 1 included Flu A, ORVs and all air quality metrics to evaluate the combined effects of environmental and viral exposures on the three defined respiratory causes of hospital admission. Model 2 further adjusted for demographic variables (sex, age and nationality) to assess whether the observed associations remained independent of individual-level characteristics. This model provided the most robust and clinically relevant prediction of ARI, capturing the combined influence of host, environmental and viral determinants. Pearson’s correlation coefficient reporting 95% CIs was calculated for respiratory admissions and confirmed respiratory virus infection using Statistics Kingdom, available at Correlation Confidence Interval Calculator (statskingdom.com).

## Results

**Fig. 1.**
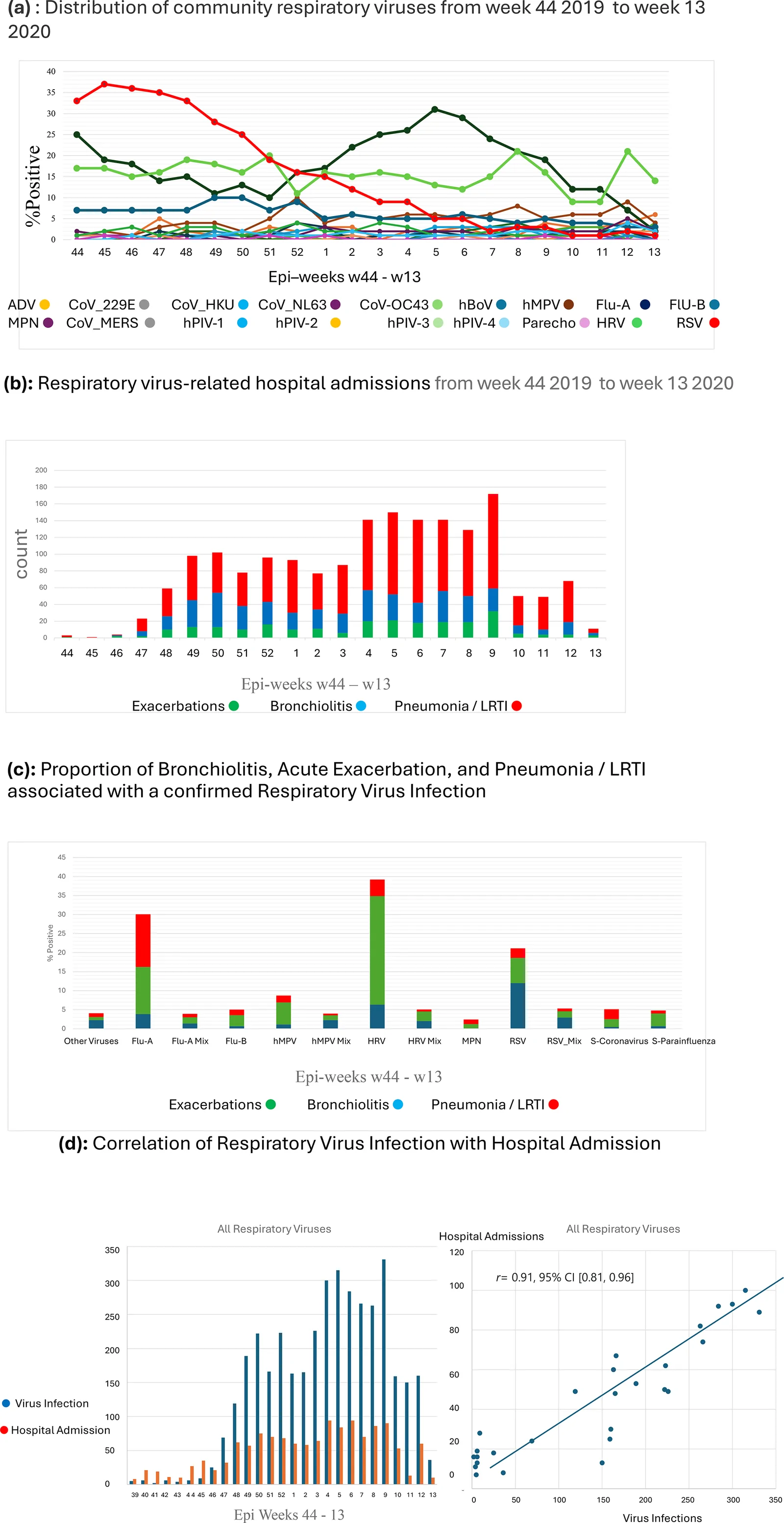
Respectively for the study period: (a) the community respiratory viral waves; (b) the hospital admissions for the three clinical conditions of pneumonia/LRTI, acute exacerbations and bronchiolitis; (c) the contribution of each respiratory virus in each clinical category; (d) the correlation of the combined acute respiratory viruses to hospital admission.

From week 44 (2019) to week 13 (2020), the distribution of respiratory viruses exhibited a typical seasonal pattern for the time of year (Fig. 1a) and was accompanied by waves of hospital admission for pneumonia, acute exacerbation and bronchiolitis (Fig. 1b). The Flu A wave peaked in week 5 (2020) at 33% of infections tested, while an RSV wave peaked in week 45 (2019) at 35% of infections tested. HRV showed moderate persistent activity throughout the period, while the remaining viruses were at baseline. Analysis of ARI by viral status revealed distinct clinical associations. Flu A was strongly linked to patients in the pneumonia group, accounting for 76.5% of cases, while ORVs were significantly more prevalent in the bronchiolitis and reactive airway groups, contributing to nearly 30% in each category (Table 1). Co-infections were uncommon.

**Table 1.** Distribution of ARIs according to viral co-detection categories (Flu A, ORVs and co-infections)

Viral co-detection category	COPD/asthma (%)	Bronchiolitis (%)	Pneumonia/ALRI (%)	Total
**Negative for both**	72 (6.4)	291 (25.9)	760 (67.7)	1,123
**Flu A only**	30 (14.7)	18 (8.8)	156 (76.5)	204
**ORV only**	134 (29.4)	135 (29.6)	187 (41)	456
**Both Flu A and ORV**	4 (22.2)	5 (27.8)	9 (50)	18
**Total**	240 (13.3)	449 (24.9)	1,112 (61.7)	1,801

This shows the distribution of single influenza A (Flu A) infections, single other respiratory virus (ORV) infections and mixed infections in each clinical category

In univariate and multivariate analyses, Flu A was identified as a risk factor for pneumonia (Table 2). Univariate analysis demonstrated that Flu A nearly doubled the odds of pneumonia admission (OR=1.932, 95% CI: 1.406–2.654, *P*<0.001). This significant association persisted after adjustment for environmental factors, with an adjusted OR (aOR=1.609, 95% CI: 1.159–2.233, *P*=0.004) in model 1, although the association was attenuated and no longer significant in the fully adjusted model (aOR=0.783, 95% CI: 0.515–1.191, *P*=0.252). Air quality metrics further modified this risk, with elevated levels of both SO_2_ and O_3_ significantly associated with increased pneumonia admissions in univariate analysis (SO_2_: OR=1.291, 95% CI: 1.062–1.596, *P*=0.01; O_3_: OR=1.285, 95% CI: 1.05–1.574, *P*=0.015). The association with SO₂ remained significant in the partially adjusted model (aOR=1.311, 95% CI: 1.044–1.646, *P*=0.02) but was attenuated and no longer significant in the fully adjusted model (aOR=1.044, 95% CI: 0.762–1.431, *P*=0.787). These associations are likely to be incidental due to the relatively short duration of the study period and the weekly averaging of the air quality data.

**Table 2.** Univariate logistic regression and multivariate logistic regression of ORVs, Flu A, air quality indicators and demographics in relation to pneumonia outcomes ORs with 95% CI are shown; *P*<0.05, age band 19–30 years as reference.

Risk factor	Univariate regression	*P*-value	Model 1	*P*-value	Model 2	*P*-value
**NO_2_>31.32**	0.989 (0.817–1.198)	0.91	0.897 (0.619–1.300)	0.566	1.075 (0.642–1.801)	0.783
**CO>0.62**	0.864 (0.714–1.045)	0.131	0.976 (0.667–1.428)	0.901	1.226 (0.728–2.065)	0.444
**NO>21.41**	0.903 (0.747–1.093)	0.295	1.220 (0.827–1.801)	0.316	0.951 (0.559–1.619)	0.854
**PM_2.5_ >27.68**	1.124 (0.93–1.36)	0.227	1.054 (0.847–1.312)	0.638	0.898 (0.664–1.215)	0.486
**O_3_>20.84**	1.285 (1.05–1.574)	**0.015**	1.355 (0.998–1.840)	0.052	1.220 (0.801–1.857)	0.354
**SO_2_>3.48**	1.291 (1.062–1.596)	**0.01**	1.311 (1.044–1.646)	**0.02**	1.044 (0.762–1.431)	0.787
**Flu A**	1.932 (1.406–2.654)	**<0.001**	1.609 (1.159–2.233)	**0.004**	0.783 (0.515–1.191)	0.252
**ORVs**	0.316 (0.255–0.393)	**<0.001**	0.333 (0.267–0.415)	**<0.001**	0.225 (0.167–0.303)	**<0.001**
**Qatari**					0.758 (0.567–1.012)	0.06
**Male**					1.042 (0.788–1.377)	0.773
**<1 year**					0.018 (0.010–0.032)	**<0.001**
**1–4 years**					0.604 (0.361–1.009)	0.054
**5–12 years**					1.998 (0.947–4.212)	0.069
**13–18 years**					3.421 (0.421–27.832)	0.25
**31–45 years**					1.872 (1.079–3.250)	**0.026**
**46–60 years**					2.177 (1.185–4.000)	**0.012**
**>60 years**					1.145 (0.688–1.905)	0.602

*Bold indicates significant *P*-value.

AQ, air quality ; CO, carbon monoxide ; Flu A, influenza A ; NO, Nitrogen Oxide ; NO_2_, nitrogen dioxide ; O_3_, ozone; OR, odds ratio; ORVs, other respiratory viruses; PM_2.5_, fine particulate matter ; SO_2_, sulfur dioxide.

In contrast, ORVs were less common among pneumonia cases in both the partially adjusted and fully adjusted models, with a markedly lower detection rate observed in pneumonia patients (partially adjusted: aOR=0.333, 95% CI: 0.267–0.415; fully adjusted: aOR=0.225, 95% CI: 0.167–0.303; *P*<0.001 for both) (Tables 2 and 3). Age stratification further demonstrated that infants <1 year had a significantly lower risk of pneumonia admission (aOR=0.018, 95% CI: 0.010–0.032, *P*<0.001), whereas adults aged 31–60 years exhibited an increased likelihood of admission (aOR=1.872, 95% CI: 1.079–3.250, *P*=0.026; aOR=2.177, 95% CI: 1.185–4.000, *P*=0.012). No significant associations were observed for gender and nationality in relation to pneumonia risk.

**Table 3.** Weekly detection frequencies of three categories of ARIs for Flu A-positive patients from epidemiological week 44 of 2019 to week 13 of 2020, stratified by demographic characteristics and corresponding air quality indices

Risk factor	Acute bronchiolitis		COPD/asthma		Pneumonia/ALRI	
	*n* (23/449)	*P*-value	*n* (34/240)	*P*-value	*n* (165/1,112)	*P*-value
**Gender**		0.553		0.714		0.641
Female (n)	10 (43.5%)		15 (44.1%)		65 (39.4%)	
Male (n)	13 (56.5%)		19 (55.9%)		100 (60.6%)	
**Nationality**		0.211		0.130		0.639
Non-Qatari (n)	11 (47.8%)		24 (70.6%)		116 (70.3%)	
Qatari (n)	12 (52.2%)		10 (29.4%)		49 (29.7%)	
**Age band**		**<0.001**		0.10		0.072
<1 year (infants)	21 (91.3%)		1 (2.9%)		3 (1.8%)	
1–4 years (pre-school)	1 (4.3%)		2 (5.9%)		23 (13.9%)	
5–12 years (school)	0		2 (5.9%)		3 (1.8%)	
13–18 years (adolescence)	0		0		3 (1.8%)	
19–30 years (young)	0		8 (23.5%)		19 (11.5%)	
31–45 years (adult)	0		9 (26.5%)		43 (26.1%)	
46–60 years (old)	1 (4.3%)		4 (11.8%)		33 (20%)	
>60 years (elderly)	0		8 (23.5%)		38 (23%)	
**AQI**		0.848		0.370		0.447
CO≤0.62	11 (47.8%)		16 (47.1%)		82 (49.7%)	
CO>0.62	12 (52.2%)		18 (52.9%)		83 (50.3%)	
		**0.016**		0.424		**<0.001**
O_3_≤0.84	21 (91.3%)		24 (70.6%)		125 (75.8%)	
O_3_>20.84	2 (8.7%)		10 (29.4%)		40 (24.2%)	
		0.216		0.953		0.098
NO≤21.41	8 (34.8%)		19 (55.9%)		77 (46.7%)	
NO>21.41	15 (65.2%)		15 (44.1%)		88 (53.3%)	
		0.795		0.598		0.596
NO_2_≤31.32	13 (56.5%)		19 (55.9%)		96 (58.2%)	
NO_2_>31.32	10 (43.5%)		15 (44.1%)		69 (41.8%)	
		0.399		0.321		**0.018**
SO_2_≤3.48	13 (56.5%)		23 (67.6%)		108 (65.5%)	
SO_2_>3.48	10 (43.5%)		11 (32.4%)		57 (34.5%)	
		0.717		0.747		0.795
PM_2.5_≤27.68	14 (60.9%)		15 (44.1%)		82 (49.7%)	
PM_2.5_>27.68	9 (39.1%)		19 (55.9%)		83 (50.3%)	
		0.316		**<0.001**		**<0.001**
ORV Pos	5 (21.7%)		4 (11.8%)		9 (5.5%)	
ORV Neg	18 (78.3%)		30 (88.2%)		156 (94.5%)	

*Bold footnote indicates *P*-value at significance <0.05.

ALRI, acute lower respiratory infection; AQI, air quality indicator ; COPD, chronic obstructive pulmonary disease; ORV, other respiratory viruses ; PM_2.5_, fine particulate matter; SO_2_, sulfur dioxide.

For bronchiolitis, Flu A had a low prevalence, with univariate analysis showing a significantly reduced risk of admission (OR=0.313, 95% CI: 0.200–0.489, *P*<0.001), and this effect remained consistent in multivariate models (aOR=0.309, 95% CI: 0.197–0.486, *P*<0.001; aOR=0.436, 95% CI: 0.200–0.953, *P*=0.038) (Tables 1 and 4). Bronchiolitis admissions were highest among infants <1 year, accounting for more than 90% of cases, with this age group demonstrating the highest associated risk (aOR=24.28, 95% CI: 15.788–37.343, *P*<0.001). Children aged 5–12 years (aOR=0.083, 95% CI: 0.020–0.351, *P*<0.001) and adults aged 46–60 years (aOR=0.018, 95% CI: 0.002–0.131, *P*<0.001) showed a significantly lower associated risk (Table 4) . Elevated levels of NO and CO were significantly associated with an increased likelihood of admission for bronchiolitis (NO: OR=1.266, 95% CI: 1.022–1.568, *P*=0.031; CO: OR=1.316, 95% CI: 1.062–1.631, *P*=0.012), whereas higher levels of O_3_, PM_2.5_ and SO_2_ were associated with a reduced likelihood of admission (O_3_: OR=0.699, 95% CI: 0.554–0.883, *P*=0.003; PM_2.5_: OR=0.745, 95% CI: 0.601–0.924, *P*=0.007; SO_2_: OR=0.736, 95% CI: 0.590–0.919, *P*=0.007). Co-infections were uncommon (Tables 3 and 4).

**Table 4.** Univariate logistic regression and multivariate logistic regression of ORVs, Flu A, air quality indicators and demographics in relation to bronchiolitis outcomes ORs with 95% CI are shown; *P*<0.05; age band 1–4 years as reference.

Risk factor	Univariate regression	*P*-value	Model 1	*P*-value	Model 2	*P*-value
**NO_2_>31.32**	1.132 (0.913–1.402)	0.258	1.191 (0.793–1.790)	0.399	0.692 (0.317–1.510)	0.355
**CO>0.62**	1.316 (1.062–1.631)	**0.012**	1.009 (0.670–1.520)	0.966	0.621 (0.285–1.352)	0.230
**NO>21.41**	1.266 (1.022–1.568)	**0.031**	0.904 (0.595–1.373)	0.635	2.420 (1.077–5.435)	**0.032**
**PM_2.5_ >27.68**	0.745 (0.601–0.924)	**0.007**	0.835 (0.656–1.063)	0.144	0.866 (0.554–1.353)	0.527
**O_3_>20.84**	0.699 (0.554–0.883)	**0.003**	0.702 (0.499–0.988)	0.060	0.665 (0.351–1.261)	0.212
**SO_2_>3.48**	0.736 (0.59–0.919)	**0.007**	0.720 (0.560–0.926)	**0.011**	1.013 (0.637–1.612)	0.956
**Flu A**	0.313 (0.2–0.489)	**<0.001**	0.309 (0.197–0.486)	**<0.001**	0.436 (0.200–0.953)	**0.038**
**ORVs**	1.381 (1.092–1.746)	**0.007**	1.229 (0.967–1.563)	0.092	0.633 (0.410–0.976)	**0.039**
**Qatari**						
**Male**						
**<1 year**					24.28 (15.788–37.343)	<0.001
**5–12 years**					0.083 (0.020–0.351)	**<0.001**
**13–18 years**					0.000	
**19–30 years**					0.000	
**31–45 years**					0.000	
**46–60 years**					0.018 (0.002–0.131)	**<0.001**
**>60 years**					0.000	

*Bold indicates significant *P*-value.

CO, carbon monoxide; Flu-A, influenza A; NO_2 _, nitrogen dioxide; NO, nitrogen oxide ; O_3 _, ozone ; OR, odds ratio; ORV, other respiratory viruses.

Flu A was associated with an increased likelihood of hospital admission for acute exacerbations across both models, with adjusted ORs ranging from 1.792 (95% CI: 1.16–2.78, *P*=0.009) to 2.020 (95% CI: 1.317–3.099, *P*=0.001) among older children and adults (Table 5). Infections in the ORV group were the strongest predictors, with a markedly elevated risk across all models for acute exacerbations (aOR=6.273, 95% CI: 4.59–8.57, *P*<0.001), reinforcing their established role in triggering airway hyperresponsiveness in asthma and reducing the surface area for oxygen transfer in COPD (Tables 1 and 5). Reflecting the inclusion of AE-asthma and AE-COPD in this group, age stratification showed increased risk across multiple age bands, respectively: children aged 1–4 years (aOR=1.834, 95% CI: 1.06–3.17, *P*=0.03), young adults 19–30 years (aOR=4.662, 95% CI: 2.64–8.25, *P*<0.001) and older adults >60 years (aOR=4.115, 95% CI: 2.61–6.61, *P*<0.001). An additional increased burden was observed among the Qatari population (aOR=1.388, 95% CI: 1.02–1.90, *P*=0.039). A breakdown of the ORV group to identify individual viruses confirmed RSV and HRV as the main viruses associated with bronchiolitis and acute exacerbations, respectively (Fig. 1c), with Flu A the commonest in patients with pneumonia. All respiratory viruses were associated with each clinical category (Fig. 1c), demonstrating a high correlation with overall admissions (Fig. 1d; *r*=0.88, 95% CI: 0.72, 0.95). Co-infections were uncommon.

**Table 5.** Univariate logistic regression and multivariate logistic regression of respiratory viruses, Flu A, air quality indicators and demographics in relation to AE-asthma and AE-COPD outcomes ORs with 95% CI are shown; *P*<0.05; age band <1 year as reference.

Risk factor	Univariate regression	*P*-value	Model 1	*P*-value	Model 2	*P*-value
**NO_2_>31.32**	0.835 (0.633–1.101)	0.202	0.912 (0.531–1.567)	0.74	1.082 (0.62–1.88)	0.781
**CO>0.62**	0.865 (0.658–1.136)	0.296	1.025 (0.592–1.774)	0.93	0.999 (0.57–1.76)	0.997
**NO>21.41**	0.839 (0.639–1.103)	0.209	0.795 (0.452–1.398)	0.426	0.716 (0.40–1.29)	0.267
**PM_2.5_>27.68**	1.265 (0.963–1.661)	0.091	1.222 (0.893–1.674)	0.211	1.203 (0.87–1.66)	0.261
**O_3_>20.84**	1.055 (0.794–1.402)	0.714	0.966 (0.634–1.474)	0.874	0.971 (0.63–1.51)	0.896
**SO_2_>3.48**	0.970 (0.735–1.279)	0.827	1.015 (0.732–1.406)	0.93	0.939 (0.67–1.32)	0.715
**Flu A**	1.205 (0.813–1.787)	0.352	2.020 (1.317–3.099)	**0.001**	1.792 (1.16–2.78)	**0.009**
**ORVs**	4.933 (3.717–6.546)	**<0.000**	5.561 (4.128–7.492)	**<0.001**	6.273 (4.59–8.57)	**<0.001**
**Qatari**					1.388 (1.02–1.90)	**0.039**
**Male**					0.982 (0.73–1.33)	0.907
**1–4 years**					1.834 (1.06–3.17)	**0.03**
**5–12 years**					1.809 (0.85–3.84)	0.123
**13–18 years**					1.491 (0.18–12.16)	0.709
**19–30 years**					4.662 (2.64–8.25)	**<0.001**
**31–45 years**					2.437 (1.45–4.10)	**<0.001**
**46–60 years**					2.008 (1.12–3.60)	**0.019**
**>60 years**					4.115 (2.61–6.61)	**<0.001**

*Bold indicates significant *P*-value.

CO, carbon monoxide; Flu-A, influenza A; NO_2_, Nitrogen Dioxide ; NO, nitrogen oxide ; O_3_, ozone ; ORV, other respiratory viruses; PM_2.5_, fine particulate matter; SO_2_, sulfur dioxide .

## Discussion

### Pneumonia and LRIs

Flu A was the commonest virus for hospital admissions in the pneumonia group followed by HRV and then seasonal coronaviruses, with smaller contributions from the other viruses; dual infections were uncommon (Fig. 1c). This was confirmed in the univariate and the partially adjusted regression model. This reflects similar reports of Flu A as a primary cause of viral pneumonia and hospitalization during seasonal outbreaks. In the fully adjusted model, inclusion of demographic variables and ORVs reduced the level of significance but the association held in the respective age bands (Table 2). Co-infections were uncommon and did not increase the risk of pneumonia, as also reported by Cebey-López *et al*. and Richard *et al*. [18, 19]. Among the air quality, only SO_2_ maintained statistical univariate significance for patients in the Flu A pneumonia group, as also reported by Kwas *et al*. and Lu *et al*. [20, 21], but this was lost in the regression model. Infants aged <12 months had a lower likelihood of admission associated with Flu A (aOR=0.018, 95% CI: 0.010–0.032, *P*<0.001). In infants aged <6 months, this may partly reflect protection from maternally derived antibodies following maternal infection or vaccination, whereas in infants aged ≥6 months, influenza vaccination may also contribute to protection; however, vaccination status was not assessed in this study. Misclassification of pneumonia as bronchiolitis in ICD-10 coding could also be a factor [22–24]. Children aged 1–18 years were also underrepresented, and again, this could be vaccine related [25, 26] or due to cross-protective immunity from previous exposures [27]; most patients in this group will not require hospitalization. In contrast, middle-aged adults (31–60 years) had a higher association with pneumonia admissions, particularly in those aged 46–60 years (aOR=2.177, 95% CI: 1.185–4.000, *P*=0.012). These observations are consistent with findings by Thompson *et al*. and Mertz *et al*. [28, 29], who identified comorbidities, smoking and obesity as relevant risk factors. This highlights middle-aged adults at increased risk of serious Flu A-associated ARI and should be the target of vaccine uptake initiatives, including in the workplace.

### Viral aetiology and bronchiolitis risk

Flu A was detected in 4% of bronchiolitis cases, with RSV (12%) the commonest followed by HRV (6%), and smaller contributions from other viruses. Mixed infections were uncommon (Fig. 1c). Across the two models, Flu A was consistently associated with ORs of <1, often reaching high levels of statistical significance, and therefore played a less important role in bronchiolitis. Children under 1 year, and under 6 months, had a high risk of hospitalization with RSV (aOR=24.28, 95% CI: 15.788–37.343, *P*<0.001). This aligns with Meissner and Shi *et al*. [30, 31], who reported that RSV accounts for 60–80% of bronchiolitis-related admissions, particularly among infants younger than 6 months. Maternal antibody would therefore appear unprotective, and combined with immature neonatal immunity, these infants are at risk of clinical infection [32, 33]. Although maternally derived antibodies against the highly conserved RSV fusion (F) glycoprotein may provide some protection in early infancy, differences in the neutralizing activity of antibodies induced by natural infection and vaccination may influence susceptibility. The observed age distribution may also reflect age-dependent immune responses to RSV infection. Support for this interpretation comes from the enhanced severity of bronchiolitis in children vaccinated against RSV [34–36] and the restriction imposed on the use of vaccines in children by the FDA [37]. Maternal immunization with a pre-F RSV vaccine and administration of neutralizing monoclonal antibodies to infants aged <8 months are effective strategies for reducing hospitalization due to severe RSV disease. These interventions currently represent important preventive measures against RSV-associated bronchiolitis, while the use of RSV vaccines in infants remains limited by safety considerations [38, 39]. Across the two models, Flu A was consistently associated with ORs of <1, often reaching high levels of statistical significance, and therefore played a less important role in bronchiolitis. Among the air quality, NO was significantly associated with bronchiolitis hospital admissions (aOR=2.420, 95% CI: 1.077–5.435, *P*=0.032), reflecting wintertime increases in NO concentrations driven by a combination of traffic-related emissions and reduced boundary layer mixing height that coincide with the RSV season. As previously reported, this association may be incidental and attributable to shared seasonal time periods rather than a direct causal effect (Husein, Poster Qatar Health Congress, 2023)[40]. The seasonal variation of NO concentrations is not independently associated with viral infection, with observed association likely explained by seasonal overlaps rather than a virus-specific mechanism. Nevertheless, NOs have been hypothesized to exert pro-inflammatory effects on the infant airway, and previous studies have reported associations between elevated NO exposure and increased hospital admissions for bronchiolitis and other LRIs in children [41, 42]. In contrast, sulphur dioxide (SO₂) showed an inverse association with bronchiolitis in unadjusted models, but the effect did not hold after adjustment for demographic and viral confounders. Other studies have demonstrated that short-term SO₂ exposure can exacerbate respiratory symptoms in children [43, 44], underscoring the complex, context-dependent nature of air quality on ARIs.

### Respiratory virus triggers in reactive airway exacerbations

HRV was the most common virus seen in acute exacerbations followed by Flu A, with smaller contributions from the other viruses. AE-asthma and AE-COPD were included in a reactive exacerbation group to reflect a similar response to minor episodes of airway inflammation, resulting in bronchospasm in asthma and altered ventilation/perfusion in COPD. This resulted in a bimodal distribution of cases across age groups. In early childhood, AE-asthma predominates, particularly among those aged 1–4 years, and the incidence of reactive airway exacerbations was significantly elevated in this age group (aOR=1.834, 95% CI: 1.06–3.17, *P*=0.03), a period when susceptibility to viral-induced wheezing is common [45, 46, 47]. A second peak of risk was observed in adults,^45^where individuals aged 19–30 years demonstrated the highest odds of reactive airway exacerbation admissions (Table 5), potentially reflecting poor adherence during the transition from paediatric to adult care [48]. Kuruvilla *et al*. [49] also noted that occupational exposures and vaping, particularly common in early adulthood, may significantly increase the risk of AE-asthma. A more detailed analysis of risk factors such as medication compliance and smoking would therefore be important but outside the scope of this study. The findings demonstrated that ORVs, in particular HRV, were strong and independent predictors of hospital admission related to acute exacerbation (aOR=6.273, 95% CI: 4.59–8.57, *P*<0.001), in line with similar reports of viral triggers of acute asthma [50, 51] (Fig. 1c). HRV is unique among respiratory viruses because of its circulation throughout the year, driven by more than 160 antigenically distinct subtypes [52, 53]. This extensive genetic diversity promotes frequent reinfections, making HRV a persistent, year-round trigger of reactive airway exacerbations in susceptible children and adults, unlike RSV and influenza, which show marked winter seasonality. Viral AE-asthma exacerbations are common throughout early childhood as a result [54], with HRV detected in 60–80% of episodes in both children and adults [52]. Notably, HRV-C has been associated with more severe wheezing and higher rates of hospitalization in preschool-aged children compared to HRV-A or HRV-B [55]. Although exacerbations typically become apparent beyond infancy, their origins in asthma often trace back to severe RSV bronchiolitis which is associated with a Th-2 immune shift [56], which may be linked to later wheezing and asthma in toddlers and preschool-aged children [57]. Adult age (31–60 years) and older age (46–60 years) patients exhibited an increased risk of hospitalization due to acute exacerbation (aOR=2.437, 95% CI: 1.45–4.10, *P*<0.001; aOR=2.008, 95% CI: 1.12–3.60, *P*=0.019, respectively), probably reflecting an increase in AE-COPD cases in the older age group. AE-asthma in older patients may also represent a mixed pathology marked by more severe symptoms and lower responsiveness to treatment [58, 59]. Furthermore, working-age individuals frequently encounter occupational exposure to dust, fumes and chemicals, which are well-recognized factors contributing to chronic lung disease [40, 60]. Studies by Gibson and Simpson and Postma and Rabe [61, 62] indicated that smoking and long-term exposure to environmental pollutants may exacerbate airway changes and lead to the development of asthma–COPD overlap (ACO). Against this background, viral infections are important with as many as 60% of acute exacerbations of COPD or ACO being associated with HRV, influenza, RSV and parainfluenza [63, 64]. Co-infections were uncommon and air quality metrics did not appear to have a significant contributory role, likely due to the short duration of the study period and the use of weekly average data to match the virology data investigations.

A further limitation is potential ICD-10 diagnostic misclassification, particularly between bronchiolitis and viral pneumonia in young children, which may have influenced the observed associations.

Although Flu A, HRV and RSV showed their strongest associations with pneumonia, reactive airway exacerbations and bronchiolitis, respectively, all respiratory viruses were represented across the three clinical presentations (Fig. 1c). Moreover, the combined respiratory-virus wave showed the strongest temporal correlation with hospital admissions (Fig. 1d), suggesting that the overall burden of circulating respiratory viruses, rather than any single pathogen, drives seasonal patterns of ARI admissions. The corresponding seasonal waves of clinical admissions (Fig. 1b) linked to these viral triggers suggest a common mechanism of action, such as an inflammatory alteration of the lung microbiome [7]. This association would encourage a focused approach to prevention, early detection and possible treatment before the need for antibiotics arises. Environmental findings should be interpreted cautiously, as weekly average air quality data may have masked short-term exposure effects. Future studies using higher-resolution exposure data and lag analyses are needed to confirm these associations.

## Conclusion

Age, patient demographics and viral type shaped the risk of acute respiratory viral diseases. The heightened inflammatory response to Flu A made it the leading cause of pneumonia, while the multiple subtypes of HRV made it the commonest cause of acute exacerbation. RSV was the main cause of bronchiolitis in children <6 months, and a partial immunopathology linked to maternal antibody remains a possible mechanism for this unique association. Importantly, all the viruses were associated with each condition. Larger, multi-season studies including SARS-CoV-2 and other circulating viruses are needed to confirm these associations across different clinical presentations.

## Abbreviations

ACO: asthma–chronic obstructive pulmonary disease overlap
ADV: Adenovirus
AE-asthma: acute exacerbation of asthma
AE-COPD: acute exacerbation of chronic obstructive pulmonary disease
aOR: adjusted odds ratio
ARI: acute respiratory infection
CI: confidence interval
CO: carbon monoxide
COPD: chronic obstructive pulmonary disease
CoV_229E: Coronavirus 229E
CoV_HKU: Coronavirus HKU1
CoV_NL63: Coronavirus NL63
CoV_OC43: Coronavirus OC43
Flu A: influenza A
Flu B: Influenza B
hBoV: Human bocavirus
hMPV: Human Metapneumovirus
hPIV-1: Parainfluenza Type 1
hPIV-2: Parainfluenza Type 2
hPIV-3: Parainfluenza Type 3
hPIV4: Parainfluenza Type 4
HRV: human rhinovirus
LRI: lower respiratory tract infection
MPN: Mycoplasma pneumonia
NO_2_: nitrogen dioxide
NO: nitrogen oxide
O_3_: ozone
OR: odds ratio
ORV: other respiratory virus
PM_2.5_: fine particulate matter
RSV: respiratory syncytial virus
RT-PCR: real-time PCR
SARS-CoV-2: severe acute respiratory syndrome coronavirus 2
SO_2_: sulphur dioxide
VIF: variance inflation factor
